# Differences in the Antigens of *Helicobacter pylori* Strains Influence on the Innate Immune Response in the *In Vitro* Experiments

**DOI:** 10.1155/2014/287531

**Published:** 2014-01-29

**Authors:** Miha Skvarc, Andreja Natasa Kopitar, Janko Kos, Natasa Obermajer, Bojan Tepes

**Affiliations:** ^1^Institute of Microbiology and Immunology, Medical Faculty Ljubljana, University of Ljubljana, Zaloska 4, 1000 Ljubljana, Slovenia; ^2^Department of Pharmaceutical Biology, Faculty of Pharmacy, University of Ljubljana, Askerceva 7, 1000 Ljubljana, Slovenia; ^3^Department of Biotechnology, Jozef Stefan Institute, Jamova 39, 1000 Ljubljana, Slovenia; ^4^AM-Diagnostic Centre Rogaska, Prvomajska ulica 29A, 3250 Rogaska Slatina, Slovenia

## Abstract

The immune response to *Helicobacter pylori* importantly determines the pathogenesis of infection as well as the success of antibiotic eradication of the bacteria. Strains of *H. pylori* were gathered from 14 patients who failed to eradicate *H. pylori* infection with antibiotics—therapy resistant strains (TRS)—or from patients who were able to eradicate *H. pylori* infection—therapy susceptible strains (TSS). The THP-1 cells were stimulated with *H. pylori* antigens. Cathepsin X expression on THP-1 cells and concentration of cytokines in the supernatant of THP-1 cells were measured with a flow cytometer. TSS *H. pylori* antigens increased the proportion of cathepsin X positive cells compared to TRS *H. pylori* antigens. TSS *H. pylori* antigens induced higher secretion of IL-12 and IL-6 compared to TRS *H. pylori* antigens (*P* < 0.001; 0.02). Polymyxin B, a lipid A inhibitor, lowered the secretion of IL-12 and IL-6 in TRS and TSS. We demonstrated a *H. pylori* strain-dependent cathepsin X and cytokine expression that can be associated with *H. pylori* resistance to eradication due to lack of effective immune response. Differences in lipid A of *H. pylori* might have an influence on the insufficient immune response, especially on phagocytosis.

## 1. Introduction

Marshall and Warren isolated *Helicobacter pylori* from gastric biopsy samples in 1982. Since then, *H. pylori* has been one of the most studied bacteria that has challenged the scientific community all over the world [1].

*H. pylori* induces chronic gastric mucosa inflammation in all infected patients, but only a minority of infected people develop peptic ulcer disease and just 1-2% of them develop gastric carcinoma. The reason for these discrepancies is to be found in the bacterial and host factors that influence the development of the disease [2].

Recent study in Slovenia estimated the prevalence of *H. pylori *to 24% [3]. The growing resistance to clarithromycin and metronidazole is responsible for the decrease of eradication rates to around 80%. Why all patients with *H. pylori *sensitive to antibiotics are not always able to eradicate *H. pylori* remains debatable [4]. One of the important host factors that affect cure rates is the immune response to the infection. *H. pylori* antigens are recognized by epithelial cells macrophages and dendritic cells with the help of Toll-like receptors (TLR) and Nod-like receptors (NLR). Activation of the above-mentioned cells leads to *H. pylori*—specific adaptive T helper type 1 response [5]. In the case of impaired host immunity, a defective immune response to *H. pylori* may be the reason for *H. pylori* eradication failure [6]. Chronic exposure to *H. pylori* can also be the result of the host's inability to induce an appropriate immune response [7].

The recent discovery of cathepsin X (CTSX) brought new knowledge that helps us understand how *H. pylori* influences the immune response [8–10]. CTSX is mainly found in the cells of the immune system of monocyte lineage, especially macrophages and dendritic cells. Higher levels of CTSX were also found in immune cells of prostate and gastric carcinomas and in macrophages of gastric mucosa infected with *H. pylori *[11, 12]. It was discovered that patients with *H. pylori *gastritis had higher cathepsin X protein and mRNA levels in gastric mucosa compared to *H. pylori *negative patients. CTSX was also upregulated in the gastric mucosa of patients with gastric cancer in comparison with those without gastric cancer [13, 14].

In our experiments, THP-1 cells, a human monocytic cell line, were used because THP-1 cells are frequently used as a model system for monocytes. They have been shown to respond with a similar transcriptional pattern as peripheral blood mononuclear cells derived macrophages after stimulation with lipopolysaccharide (LPS) from *Escherichia coli *[15, 16]. THP-1 cells were also used in the experiments where it was proposed that CTSX mediates activation of *β*-2 integrin receptor Mac-1 and suppresses the stimulatory signal in the form of cytokines in the patients that failed to eradicate the *H. pylori* [17]. Results from German study suggest that *H. pylori* induced overexpression of CTSX in macrophages and epithelium through specific cytokines that are initiated by CagA-dependent pathways in a cell type-dependent manner [18].

The aim of this study was to determine the role of CTSX in the immune response to *H. pylori*. We were interested in the differences in the expression of CTSX and cytokine immune response if we stimulate immune cells with *H. pylori* from patients that are not capable of eradicating the infection, in contrast to *H. pylori* from patients who should be able to eradicate *H. pylori*.

## 2. Materials and Methods

### 2.1. Patients

We included 14 strains of *H. pylori* isolated from 14 dyspeptic patients. All patients needed reevaluation after unsuccessful eradication therapy. Seven patients had *H. pylori *sensitive to all antibiotics at the reevaluation and have failed to eradicate the bacteria because they were not able to follow treating regime. We used the strains that were isolated from gastric biopsies taken at initial gastroscopy and named them therapy sensitive group—TSS group. All of these patients eradicated bacteria after second try with the same therapy and the same regime as in the first unsuccessful attempt. Seven patients failed to eradicate the bacteria although they followed advice of a gastroenterologist, how to take the therapy. The strains were again sensitive to all antibiotics that were used in the first attempt to eradicate *H. pylori. *We used strains of *H. pylori *isolated from biopsies taken at their first gastroscopy and named them therapy resistant group—TRS group. These patients failed to eradicate the bacteria till their therapy was switched from amoxicillin plus clarithromycin to amoxicillin plus metronidazole for 14 days. The switch to the eventually successful therapy was done after second unsuccessful treatment in 5 patients and after third unsuccessful treatment in 2 patients. In these 2 patients *H. pylori *developed clarithromycin resistance.

All patients were well informed about the study, they agreed to have additional gastroscopies, and they documented antibiotic use by specifying day and time of every drug intake. The study was approved by the National Medical Ethics Committee of the Ministry of Health of the Republic of Slovenia, and written consent was obtained from patients before specimen collection.

### 2.2. *H. pylori* Culturing and Antigen Preparation

Gastric mucosa biopsies from patients were inoculated onto BHI agar with 10% of horse blood agar and spread over the surface for 3–5 days to obtain *H. pylori* strains. Antibiotic resistance and minimal inhibitory concentration (MIC) were determined by *E*-test. MIC for metronidazole was tested with agar dilution method. The breakpoints were set according to CLSI and EUCAST standards. Antibiotic susceptibility testing was done on Mueller Hinton agar with old sheep blood. Antibiotic resistance was tested on all isolates. Typical bacterial colonies were collected, suspended in phosphate buffered saline (PBS), pH 7.4, and sonicated to provide antigens with Misonix Sonificator LX. Total protein concentration was determined by Bio-Rad protein Assay (Bio-Rad, USA) and was adjusted to 10.0 mg/mL. Suspensions of bacterial antigens were filtered through 0.45 *μ*m membrane filters and stored at −20°C until use.

### 2.3. Culture of THP-1 Cells

Advanced RPMI 1640 supplemented with 2 mM L-glutamine, 5% FCS (Hyclone, Logan, UT, USA), and antibiotics (penicillin and streptomycin) (Sigma, St. Louis, MO, USA) were added to THP-1 cells (TIB-202, LGC Promochem, UK) and cultured for 7 days.

### 2.4. Stimulation of THP-1 Cells with *H. pylori* Antigens

THP-1 cells were adjusted to a final concentration of 10^6^ cells/mL and 900 *μ*L of cell suspension was added to a 24-well plate (Corning Costar, USA). 100 *μ*L of different bacterial antigen suspension was added. Cells were incubated in the presence of *H. pylori* antigens for 48 h at 37°C. The plate was then placed on ice and the cells were harvested with ice-cold PBS. Cells were centrifuged at 2000 rpm for 5 min and labelled with anti-HLA-DR IgG-PE (Becton Dickinson, USA) and anti-cathepsin X mAb-Alexa 488 (Faculty of Pharmacy, Ljubljana, Slovenia) [19]. They were washed with PBS and analysed on the flow cytometer (FACS Canto II, Becton Dickinson, USA). In the negative control sample, the antigen was not added to the cells.

### 2.5. Measurement of Cytokines Concentrations in the Supernatant of *H. pylori* Primed THP-1 Cells

We defrosted the *H. pylori* antigens and prepared THP-1 cells to a final concentration of 10^6^ cells/mL. A 900 *μ*L of cell suspension was added to a 24-well plate (Corning Costar, USA). We added 100 *μ*L of each antigen with or without 10 µg/mL of polymyxin B to THP-1 cells. Cells were incubated in the presence of antigen and in the presence of antigens and polymyxin B for 48 h at 37°C. The cytokines in the supernatant were measured with the help of BD CBA Flex Set as suggested in the manufacturer's manual (Becton Dickinson, USA). We analysed the sample on the flow cytometer (FACS Canto II, Becton Dickinson, USA). In the negative control sample, the antigens and polymyxin B were not added to the cells.

### 2.6. Statistical Analysis

All the experiments were done in triplicate and the mean values of the measured expression of CTSX and cytokines were calculated and used in statistical analysis. Differences between study groups were analysed using the unpaired Student's *t*-test, and *P* < 0.05 was taken as significant. Differences between the concentrations of cytokines of the two study groups were analysed with Mann-Whitney test, and *P* < 0.05 was taken as significant. All the calculations were done with SPSS PASW Statistics 18 programme.

## 3. Results 

**Figure 1:**
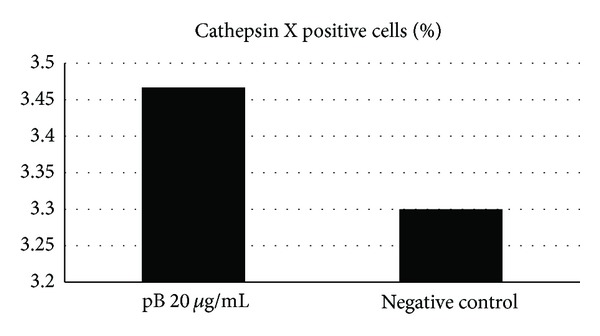
Expression of cathepsin X on THP-1 cells after stimulation with polymyxin B (pB) and when antigens of *H. pylori *were not added to THP-1 cells (negative control).

We measured the number of THP-1 cells stimulated with *H. pylori* antigens that expressed CTSX. TSS *H. pylori* antigens increased the proportion of CTSX positive cells compared to TRS* H. pylori* antigens (Table 1). The difference was not statistically significant. When we repeated the experiments several times, the difference became statistically significant. Less THP-1 cells expressed CTSX on its membrane if they were stimulated with TRS strains (data not shown). Analysis of proportion of CTSX positive THP-1 cells in *H. pylori* stimulated THP-1 revealed an increased proportion of CTSX positive cells compared to unstimulated negative control THP-1 cells. The difference was statistically significant (*P* < 0.009, Figure 1).

**Figure 2:**
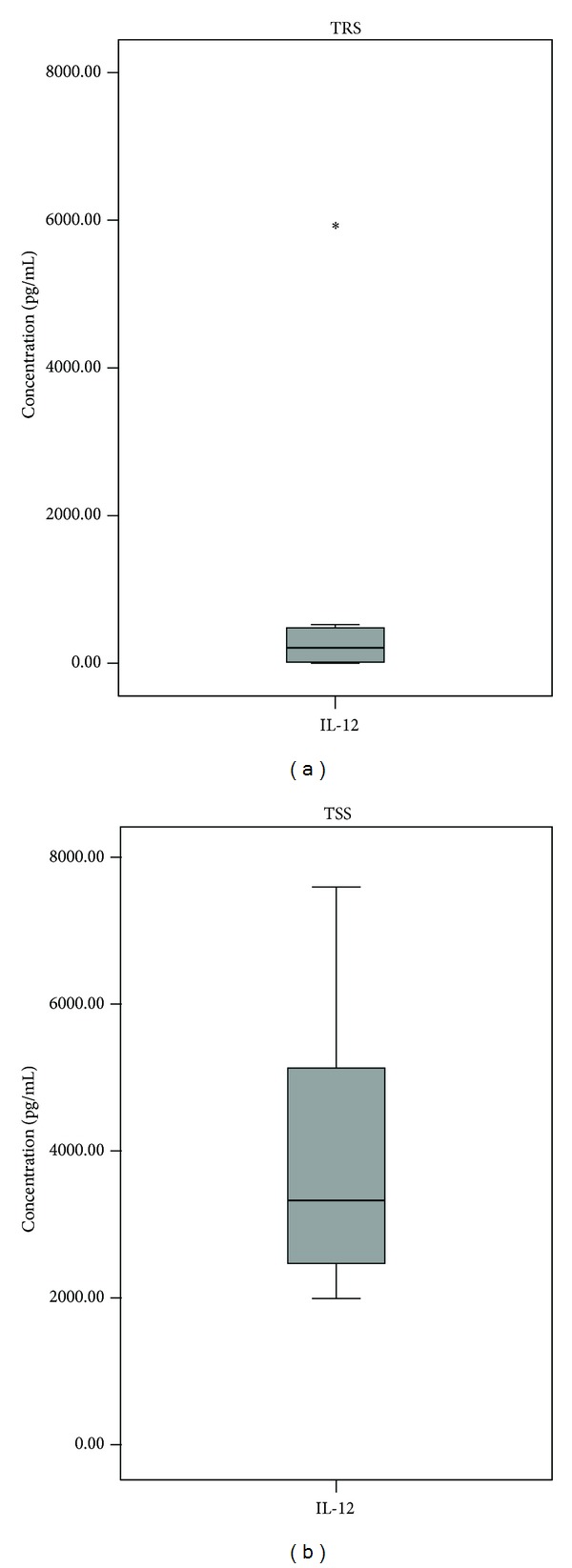
The concentration of IL-12 in pg/mL when THP-1 cells were stimulated with *H. pylori* therapy resistant strains (TRS) or *H. pylori* therapy susceptible strains (TSS). The difference is statistically significant.

We measured the *in vitro* secretion of IL-12, IL-6, IL-17, IFN-*γ*, and IL-10 by THP-1 cells stimulated with TRS or TSS *H. pylori* antigens. In the negative control sample (supernatants of unstimulated THP-1 cells), we did not detect any of the above-mentioned cytokines. The same occurred when we measured cytokines in the supernatant, where the THP-1 cells were stimulated only with polymyxin B. After stimulating THP-1 cells with TRS or TRR *H. pylori* antigens, only IL-12 and IL-6 were produced above the assay detection limit level. The concentrations of IL-12 and IL-6 were much higher in TSS group than in the TRS group (Table 2, Figure 2). Polymyxin B, a lipid A inhibitor, lowered the secretion of IL-12 and IL-6 in TRS and TSS in comparison to experiment without polymyxin B. The concentrations of both cytokines in the TRS group were below detection limits of the assay; however, the secretion of IL-6 and especially IL-12 remained high in the TSS group (Table 3).

## 4. Discussion

The failure of immune system to eradicate *H. pylori* during the acute phase of infection may result in an inadequate immune response leading to chronic gastritis, development of peptic ulcer disease, or even gastric cancer [20]. We have proven in our previous study that the success of *H. pylori* eradication therapy by antibiotics is at least partially dependent upon cytokine response by T cells [19]. Obermajer et al. studied the expression of CTSX and also used strain of *H. pylori *from individuals that had problems with eradication of bacteria. They discovered higher membrane expression of CTSX in stimulated monocytes, derived from buffy coat of blood donors, after stimulation with *H. pylori* strains from those subjects who did not respond to antibiotic therapy [17]. Strains included in our study also had a specific clinical outcome of *H. pylori* eradication therapy. These strains induced expression of CTSX on the membrane of stimulated THP-1 cells. We found more THP-1 cells expressing CTSX, being higher after stimulation with *H. pylori* gathered from those subjects who responded to antibiotic therapy in comparison to TRS group although the difference was not significant. But THP-1 cells stimulated with TSS strains produced significantly more IL-12 in comparison to TRS strains. We suspect that the level of cytokine response was much stronger *in vivo* in those patients that eventually eradicate the bacteria. The differences between the study of Obermajer et al. [17] and our study happened because they used monocytes derived from buffy coat and the influence of possible individual variances in the immune response could not be excluded.

It was proposed that VacA protein of *H. pylori *efficiently enters activated, migrating primary human T lymphocytes by binding to the *β*-2 (CD18) integrin receptor subunit and exploiting the recycling of LFA-1 [21]. It was demonstrated that another protein HP-NAP is able to cross the endothelium and stimulate polymorphonuclear cells to adhere *in vivo*, in underflow conditions. This effect is directly mediated by HP-NAP, which induces a high affinity state of *β*-2 integrin on polymorphonuclear cells [22, 23]. For appropriate action of LFA-1, CTSX is essential. CTSX has been shown to regulate T-cell migration by interaction with LFA-1. It gradually cleaves the C-terminal of the *β*-2 cytoplasmic tail of LFA-1 and enables the transition between intermediate and high affinity LFA-1, an event that is crucial for effective T-cell migration [24].

In the present study, we demonstrated that failure of *H. pylori* eradication may be due to inability of innate immune response to clear the infection. When polymyxin B was added to the THP-1 cells primed with *H. pylori *antigen, the levels of IL-12 and IL-6 were lower in the group strains that were able to persist in the stomach despite appropriate antibiotic therapy. Apparently, lipid A, to which polymyxin B binds, is an important factor that has a role in avoiding a strong immune response.

LPS, composed of a lipid A, is an important molecule in the initiation of an immune response. Data suggest that different LPS variants exist in the colonizing *H. pylori* population, which can adapt to changes in the gastric environment and provide a means of regulating the inflammatory response of the host during disease progression [25]. LPS diversity within the subpopulation of *H. pylori *is important in regulating the host's inflammatory response. LPS variants have a different influence on the cytokine level and on the innate immune response and clearance of the infection [26].

We have proven in our recent study that some strains of *H. pylori *differ in their capability to induce dendritic cells maturation and antigen-presenting function. When we inhibited the action of CTSX, we noticed reduced secretion of IL-6 and the secretion was significantly lower in the group of *H. pylori* strains isolated from patients with repeated antibiotic eradication failure [27]. We have also proven that inhibition of CTSX influences the internalization of Toll-like receptors 2 and 4. The beginning of a successful immune response against *H. pylori* in the case of CTSX inhibition is delayed [28].

## 5. Conclusion

We suggest that when bacteria *H. pylori* are engulfed by macrophages differences in *H. pylori*, lipopolysaccharide structure prevents an efficient innate immune response. Some strains of *H. pylori* are able to influence CTSX which is important protein that enables sufficient fluidity of the membrane of antigen presenting cells which are vital for the beginning of successful immune response and eradication of *H. pylori*.

## Figures and Tables

**Table 1:** Expression of CTSX on THP-1 cells after stimulation with different *H. pylori* antigens.

	Therapy resistant strains* (TRS)	Therapy susceptible strains* (TSS)	*P* value
% THP-1 with expressed CTSX	7.24 (SD ± 10.47)	13.26 (SD ± 5.29)	0.2

*TRS group (7 strains) and TSS group (7 strains); SD: standard deviation. To compare means we used independent sample *t-*test. CTSX: cathepsin X.

**Table 2:** Concentration of cytokines^&^ in supernatant of THP-1 cells stimulated with different *H. pylori* antigens.

	Therapy resistant strains* (TRS)	Therapy susceptible strains* (TSS)	Median difference (95% CI )	*P* value
IL-12 (pg/mL)	209	3359	2089 (403–4195)	0.001
IL-6 (pg/mL)	0	57	0 (0–110)	0.02

*TRS group (7 strains) and TSS group (7 strains). ^&^Concentrations in medians. To compare medians we used Mann-Whitney *U* test.

**Table 3:** Concentration of cytokines^& ^ in supernatant of THP-1 cells stimulated with different *H. pylori* antigens and with polymyxin B.

	Therapy resistant strains* (TRS)	Therapy susceptible strains* (TSS)	Median difference (95% confidence interval )	*P* value
IL-12 (pg/mL)	0	425	115 (0–456)	0.001
IL-6 (pg/mL)	0	9.25	0 (0–19)	0.01

*TRS group (7 strains) and TSS group (7 strains). ^&^Concentrations in medians. To compare medians we used Mann-Whitney *U* test.
